# Bis-naphtho-γ-pyrones from Fungi and Their Bioactivities

**DOI:** 10.3390/molecules19067169

**Published:** 2014-05-30

**Authors:** Shiqiong Lu, Jin Tian, Weibo Sun, Jiajia Meng, Xiaohan Wang, Xiaoxiang Fu, Ali Wang, Daowan Lai, Yang Liu, Ligang Zhou

**Affiliations:** 1 MOA Key Laboratory of Plant Pathology, Department of Plant Pathology, College of Agronomy and Biotechnology, China Agricultural University, Beijing 100193, China; E-Mails: shiqionglu@126.com (S.L.); pstianjin@cau.edu.cn (J.T.); sunweibo.1001@163.com (W.S.); mengjiajiax@163.com (J.M.); wangxiaohan99@126.com (X.W.); xiaoxiaofu@cau.edu.cn (X.F.); wangali526@163.com (A.W.); dwlai@cau.edu.cn (D.L.); 2 Institute of Agro-products Processing Science and Technology, Chinese Academy of Agricultural Sciences, Beijing 100193, China

**Keywords:** bis-naphtho-γ-pyrones, chaetochromin, asperpyrone, nigerone, fungi, biological activities

## Abstract

Bis-naphtho-γ-pyrones are an important group of aromatic polyketides derived from fungi. They have a variety of biological activities including cytotoxic, antitumor, antimicrobial, tyrosine kinase and HIV-1 integrase inhibition properties, demonstrating their potential applications in medicine and agriculture. At least 59 bis-naphtho-γ-pyrones from fungi have been reported in the past few decades. This mini-review aims to briefly summarize their occurrence, biosynthesis, and structure, as well as their biological activities. Some considerations regarding to synthesis, production, and medicinal and agricultural applications of bis-naphtho-γ-pyrones are also discussed.

## 1. Introduction

Bis-naphtho-γ-pyrones (also known as dimeric naphtho-γ-pyrones, bis(naphtho-γ-pyrone)s, or bis-naphthopyran-4-ones) are an important group of fungal polyketides [1]. The interest of many investigators in this class of compounds is due to their broad-range biological activities with potential applications in medicine and agriculture [2,3,4]. Until now, fungal bis-naphtho-γ-pyrones and their biological activities have not been reviewed. This comprehensive mini-review describes the occurrence, biosynthesis, and biological activities of fungal bis-naphtho-γ-pyrones. We also discuss their synthesis, production and applications.

## 2. Occurrence

**Figure 1:**
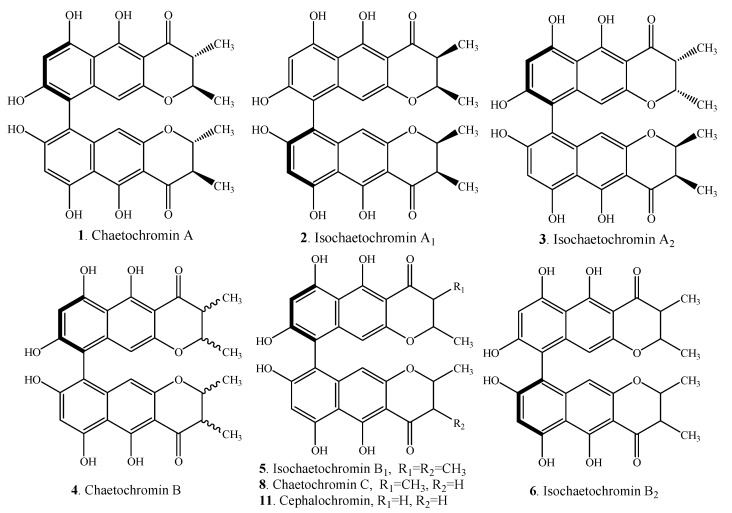

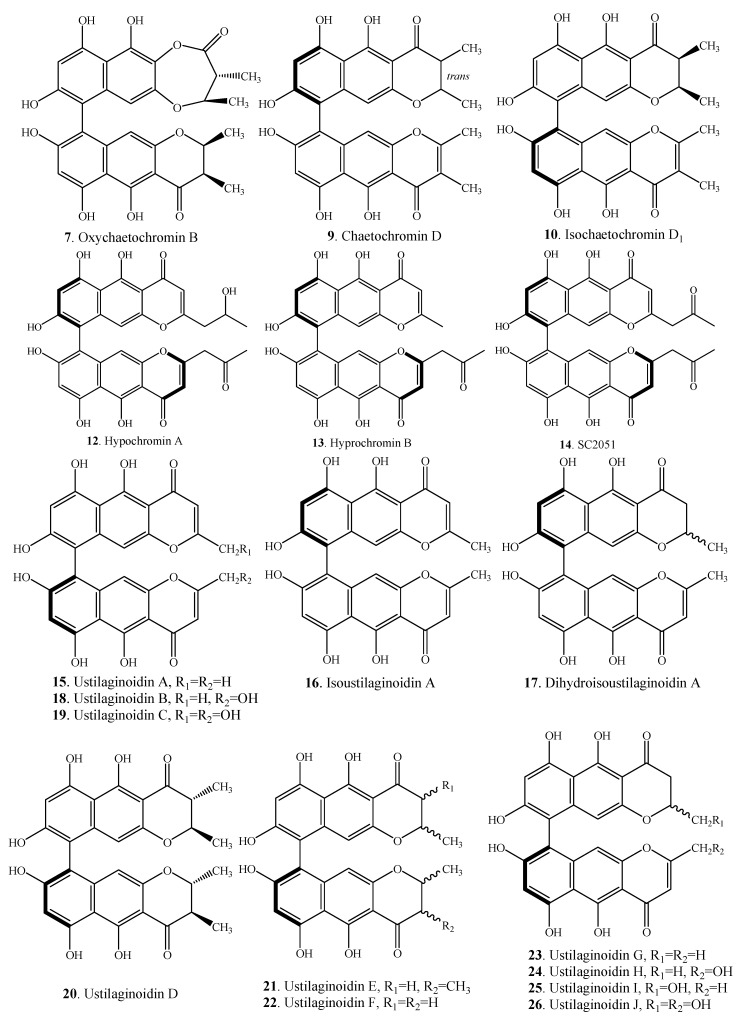
Structures of chaetochromin-type bis-naphtho-γ-pyrones (**1**–**26**) from fungi.

**Figure 2:**
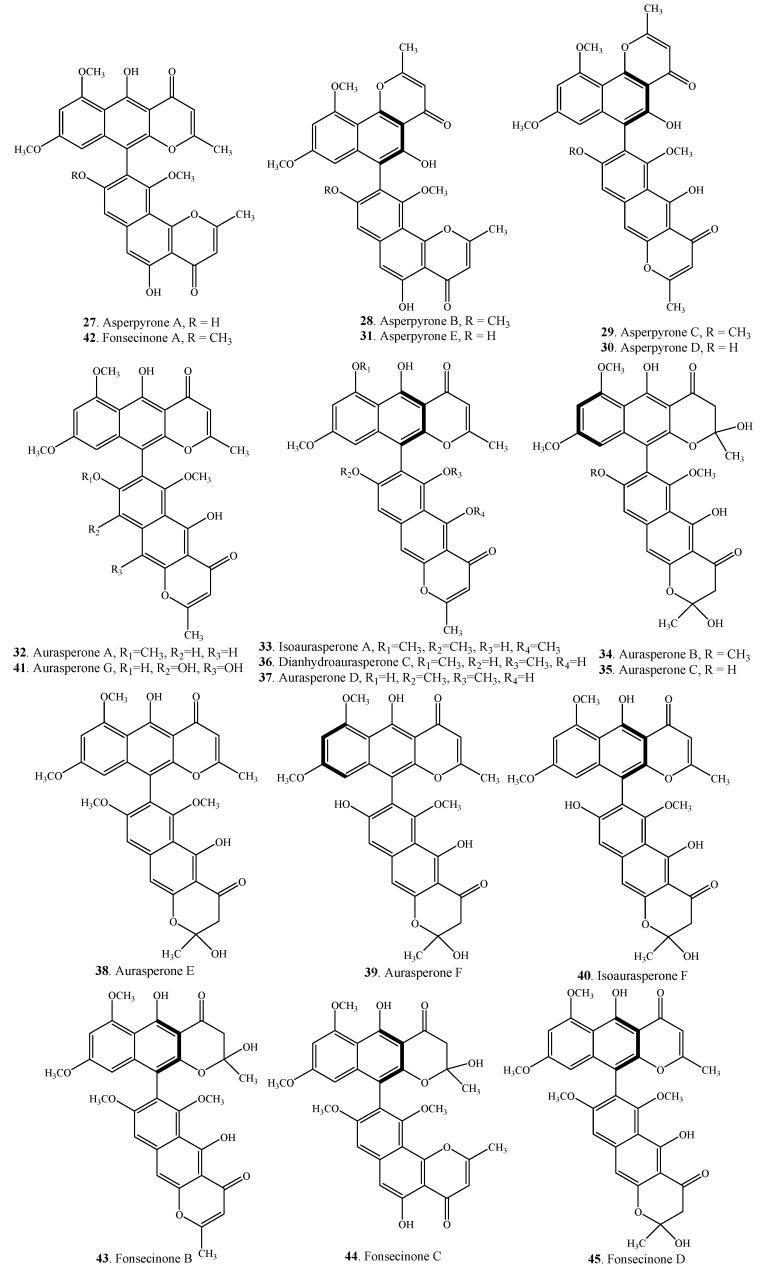

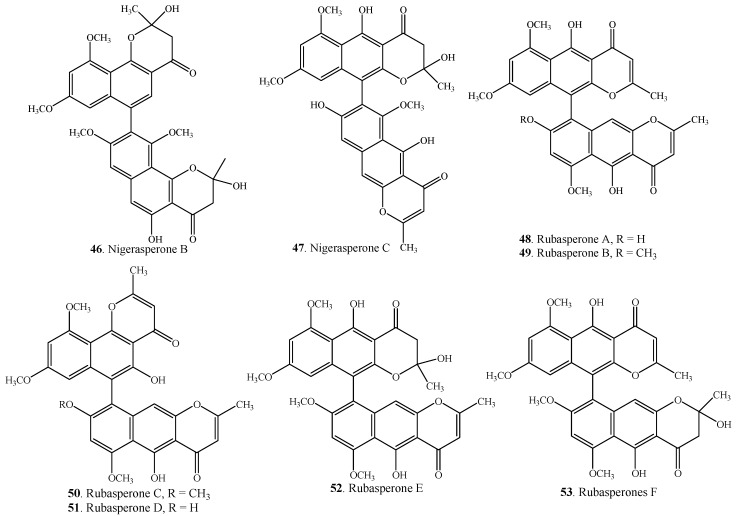
Structures of asperpyrone-type bis-naphtho-γ-pyrones (**27**–**53**) from fungi.

**Figure 3:**
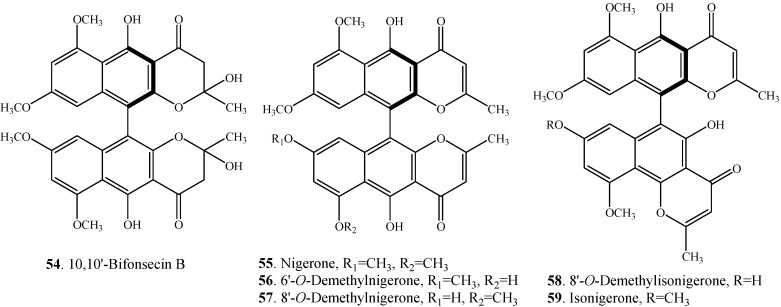
Structures of nigerone-type bis-naphtho-γ-pyrones (**54**–**59**) from fungi.

Bis-naphtho-γ-pyrones have a diverse distribution in fungi (Table 1, Table 2 and Table 3). Their structures are shown in Figure 1, Figure 2 and Figure 3. Based on the diaryl bond connection, bis-naphtho-γ-pyrones can be divided into three types (or groups), namely chaetochromin-, asperpyrone-, and nigerone-type. The absolute configurations of dimeric naphtho-γ-pyrones have been determined by circular dichroism (CD), 2D-NMR as well as X-ray diffraction analysis of some derivatives [5,6,7]. According to the literature, *R*-configured dimeric naphtho-γ-pyrones exhibit a first negative Cotton effect in the long-wavelength region, and a positive second one at shorter wavelength. On the contrary, *S*-configured dimeric naphtho-γ-pyrones exhibit a positive Cotton effect first in the long-wavelength region, and a negative one second at shorter wavelength [6,8].

To date, twenty-six chaetochromin-type bis-naphtho-γ-pyrones (Table 1 and Figure 1) with C-9-C-9' linkages have been isolated from the genera *Chaetomium*, *Hypocrea*, *Nectria*, *Penicillium*, *Verticillium*, and *Villosiclava* (*Ustilaginoidea*). The absolute configurations of ustilaginoidin A (**15**) and the congeners from *Ustilaginoidea virens* were proved to be *R*, and the congeners of chaetochromin A (**1**) from *Chaetomium* spp. were *S* [9]. Both isochaetochromins B_1_ (**5**) and B_2_ (**6**) from *Fusarium* sp., *Penicillium* sp. and *Metarhizium anisopliae* were considered as the diastereomers of chaetochromin B (**4**) [10].

**Table 1:** Occurrence of chaetochromin-type bis-naphtho-γ-pyrones **1**–**26** in fungi.

Bis-naphtho-γ-pyrone	Fungal Species	Reference
Chaetochromin A (**1**)	Endophytic fungus *Chaetomium chiversii*	[11]
	*Chaetomium gracile*	[10]
	*Chaetomium microcephalum*	[12]
	*Chaetomium virenscens* (*C. thielavioideum*)	[13]
Isochaetochromin A_1_ (**2**)	*Penicillium* sp. FK I-4942	[14,15]
Isochaetochromin A_2_ (**3**)	*Chaetomium microcephalum*	[12]
Chaetochromin B (**4**)	*Chaetomium gracile*	[6,10]
	*Chaetomium microcephalum*	[12]
Isochaetochromin B_1_ (**5**)	Endophytic fungus *Fusarium* sp.	[3]
	*Penicillium* sp. FKI-4942	[14]
Isochaetochromin B_2_ (**6**)	Endophytic fungus *Fusarium* sp.	[3]
	Sponge-derived fungus *Metarhizium anisopliae*	[16]
	*Penicillium* sp. FKI-4942	[14]
Oxychaetochromin B (**7**)	Endophytic fungus *Fusarium* sp.	[3]
Chaetochromin C (**8**)	*Chaetomium gracile*	[10]
Chaetochromin D (**9**)	*Chaetomium gracile*	[10]
Isochaetochromin D_1_ (**10**)	Endophytic fungus *Fusarium* sp.	[3]
Cephalochromin = Sch 45752 (**11**)	*Cephalosporium* sp.	[17]
	*Cosmospora vilior* YMJ89051501	[18]
	*Nectria flavoviridis*	[19]
	*Nectria viridescens*	[19]
	Endophytic fungus *Pseudoanguillospora* sp.	[20]
	*Verticillium* sp. K-113	[21]
	Unidentified fungal isolate SCF-125	[22]
Hypochromin A (**12**)	Marine-derived fungus *Hypocrea vinosa*	[23]
Hypochromin B (**13**)	Marine-derived fungus *Hypocrea vinosa*	[23]
SC2051 (**14**)	Marine-derived fungus *Hypocrea vinosa*	[23]
Ustilaginoidin A (**15**)	*Villosiclava virens* (*Ustilaginoidea virens*)	[24]
Isoustilaginoidin A (**16**)	*Verticillium* sp. K-113	[21]
Dihydroisoustilaginoidin A (**17**)	*Verticillium* sp. K-113	[21]
	Unidentified fungal isolate SCF-125	[22]
Ustilaginoidin B (**18**)	*Villosiclava virens* (*Ustilaginoidea virens*)	[25]
Ustilaginoidin C (**19**)	*Villosiclava virens* (*Ustilaginoidea virens*)	[25]
Ustilaginoidin D (**20**)	Sponge-derived fungus *Metarhizium anisopliae*	[16]
	*Villosiclava virens* (*Ustilaginoidea virens*)	[26]
Ustilaginoidin E (**21**)	*Villosiclava virens* (*Ustilaginoidea virens*)	[26]
Ustilaginoidin F (**22**)	*Villosiclava virens* (*Ustilaginoidea virens*)	[26]
Ustilaginoidin G (**23**)	*Villosiclava virens* (*Ustilaginoidea virens*)	[26]
Ustilaginoidin H (**24**)	*Villosiclava virens* (*Ustilaginoidea virens*)	[26]
Ustilaginoidin I (**25**)	*Villosiclava virens* (*Ustilaginoidea virens*)	[26]
Ustilaginoidin J (**26**)	*Villosiclava virens* (*Ustilaginoidea virens*)	[26]

Twenty-seven asperpyrone-type bis-naphtho-γ-pyrones (Table 2 and Figure 2) with C-10-C-7' or C-10-C-9' or C-6-C-7' or C-6-C-9' linkages have been isolated from the genera *Alternaria* and *Aspergillus*.

Aurasperone A (**32**) is a 10,7'-bisnaphtho-γ-pyrone from *Alternaria alternata* [27] and a few *Aspergillus* species [4,28,29,30,31,32,33]. This compound clearly has a positive Cotton effect first in the long wavelength region, and a negative one second at shorter wavelength, indicating positive chirality of the chromophores and the *S*-configuration of the compound [6]. The absolute configurations for some asperpyrone-type bis-naphtho-γ-pyrones remain to be determined.

**Table 2:** Occurrence of asperpyrone-type bis-naphtho-γ-pyrones **27**–**53** in fungi.

Bis-naphtho-γ-pyrone	Fungal Species	Reference
Asperpyrone A (**27**)	Endophytic fungus *Aspergillus* sp.	[34]
	Endophytic fungus *Aspergillus* sp. DCS31	[35]
	*Aspergillus niger*	[30]
	Endophytic fungus *Aspergillus niger*	[8]
	Endophytic fungus *Aspergillus tubingensis*	[36]
Asperpyrone B (**28**)	*Aspergillus niger*	[30]
	*Aspergillus niger* IFB-E003	[31]
	*Aspergillus niger* ATCC 11414	[33]
Asperpyrone C (**29**)	*Aspergillus niger*	[30]
Asperpyrone D (**30**)	Endophytic fungus *Aspergillus* sp. DCS31	[35]
	Endophytic fungus *Aspergillus niger*	[8]
	Endophytic fungus *Aspergillus tubingensis*	[36]
Asperpyrone E (**31**)	Endophytic fungus *Aspergillus niger*	[8]
Aurasperone A (**32**)	*Alternaria alternata*	[27]
	*Aspergillus* sp. FKI-3451	[4]
	*Aspergillus awamori*	[28,37]
	Endophytic fungus *Aspergillus auleatus*	[32]
	*Aspergillus fonsecaeus*	[29]
	*Aspergillus niger*	[28,37]
	*Aspergillus niger*	[38]
	*Aspergillus niger*	[30]
	*Aspergillus niger* IFB-E003	[31]
	*Aspergillus niger* ATCC 11414	[33]
	Endophytic fungus *Aspergillus tubingensis*	[36]
Isoaurasperone A (**33**)	Endophytic fungus *Aspergillus* sp.	[34]
	*Aspergillus niger*	[38]
	Endophytic fungus *Aspergillus niger*	[8]
Aurasperone B (**34**)	*Alternaria alternata*	[27]
	*Aspergillus* sp. FKI-3451	[4]
	*Aspergillus awamori*	[28,39]
	*Aspergillus fonsecaeus*	[29]
	*Aspergillus niger*	[28,39]
	*Aspergillus niger*	[38]
	*Aspergillus niger* ATCC 11414	[33]
	*Aspergillus niger* C-433	[40]
Aurasperone B (**34**)	*Aspergillus niger* ATCC 11414	[33]
	*Aspergillus vadensis*	[41]
Aurasperone C (**35**)	*Alternaria alternata*	[27]
	*Aspergillus awamori*	[28,39]
	*Aspergillus niger*	[28,39]
	*Aspergillus niger*	[38]
	*Aspergillus niger* C-433	[40]
	*Aspergillus niger* ATCC 11414	[33]
Dianhydro-aurasperone C (**36**)	Endophytic fungus *Aspergillus* sp.	[34]
	*Aspergillus niger*	[38]
	Endophytic fungus *Aspergillus niger*	[8]
	Endophytic fungus *Aspergillus tubingensis*	[36]
Aurasperone D (**37**)	*Aspergillus niger*	[42]
	*Aspergillus niger*	[38]
	Endophytic fungus *Aspergillus niger*	[8]
	*Aspergillus niger* C-433	[40]
Aurasperone E (**38**)	*Aspergillus niger*	[38]
	*Aspergillus niger* C-433	[40]
	Endophytic fungus *Aspergillus tubingensis*	[36]
Aurasperone F (**39**)	*Alternaria alternata*	[27]
	*Aspergillus niger* C-433	[40,43]
Isoaurasperone F (**40**)	Endophytic fungus *Aspergillus niger*	[8]
Aurasperone G (**41**)	*Aspergillus niger* C-433	[43]
Fonsecinone A (**42**)	Endophytic fungus *Aspergillus* sp.	[34]
	Endophytic fungus *Aspergillus auleatus*	[32]
	*Aspergillus fonsecaeus*	[29]
	*Aspergillus niger*	[30]
	*Aspergillus niger* IFB-E003	[31]
	*Aspergillus niger* ATCC 11414	[33]
	Endophytic fungus *Aspergillus tubingensis*	[36]
	Endophytic fungus *Cladosporium herbarum* IFB-E002	[44]
Fonsecinone B (**43**)	*Aspergillus fonsecaeus*	[29]
	*Aspergillus niger* ATCC 11414	[33]
Fonsecinone C (**44**)	*Aspergillus fonsecaeus*	[29]
	*Aspergillus niger* ATCC 11414	[33]
Fonsecinone D (**45**)	*Aspergillus fonsecaeus*	[29]
Nigerasperone B (**46**)	*Aspergillus niger* EN-13	[45]
Nigerasperone C (**47**)	*Aspergillus niger* EN-13	[45]
Rubasperone A (**48**)	Endophytic fungus *Aspergillus tubingensis*	[46]
Rubasperone B (**49**)	Endophytic fungus *Aspergillus tubingensis*	[46]
Rubasperone C (**50**)	Endophytic fungus *Aspergillus tubingensis*	[46]
Rubasperone D (**51**)	Endophytic fungus *Aspergillus tubingensis* (GX1-5E)	[47]
Rubasperone E (**52**)	Endophytic fungus *Aspergillus tubingensis* (GX1-5E)	[47]
Rubasperone F (**53**)	Endophytic fungus *Aspergillus tubingensis* (GX1-5E)	[47]

Six nigerone-type bis-naphtho-γ-pyrones (**54**–**59**) with C-10-C-10' or C-10-C-6' linkages have been isolated from the genus *Aspergillus* [5,7]. All these bis-naphtho-γ-pyrones have *R*-configurations of the 10-10' or 10-6' bonds.

It is worth mentioning that both asperpyrone and nigerone types of bis-naphtho-γ-pyrones are produced primarily by *Aspergillus* species where chaetochromin-type bis-naphtho-γ-pyrones do not distribute. This indicates that bis-naphtho-γ-pyrones should have taxonomic significance which needs to be further investigated [41]. Each fungal species also needs to be clearly identified [48,49,50].

**Table 3:** Occurrence of nigerone-type bis-naphtho-γ-pyrones **54**–**59** in fungi.

Bis-naphtho-γ-pyrone	Fungal Species	Reference
10,10'-Bifonsecin B (**54**)	Marine-derived fungus *Aspergillus carbonarius*	[7]
Nigerone (**55**)	Marine-derived fungus *Aspergillus carbonarius*	[7]
	*Aspergillus niger*	[5]
6'-*O*-Demethylnigerone (**56**)	Marine-derived fungus *Aspergillus carbonarius*	[7]
	*Aspergillus niger*	[5]
8'-*O*-Demethylnigerone (**57**)	Marine-derived fungus *Aspergillus carbonarius*	[7]
8'-*O*-Demethylisonigerone (**58**)	Marine-derived fungus *Aspergillus carbonarius*	[7]
Isonigerone (**59**)	Marine-derived fungus *Aspergillus carbonarius*	[7]
	*Aspergillus niger*	[5]

## 3. Biosynthesis

**Scheme 1:**
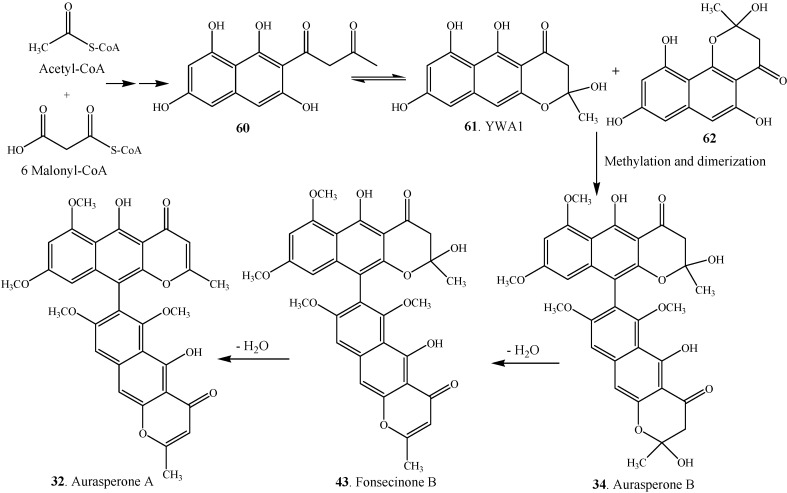
Proposed biosynthetic pathway of asperpyrone-type bis-naphtho-γ-pyrones in *Aspergillus niger* [33].

**Figure 4:**
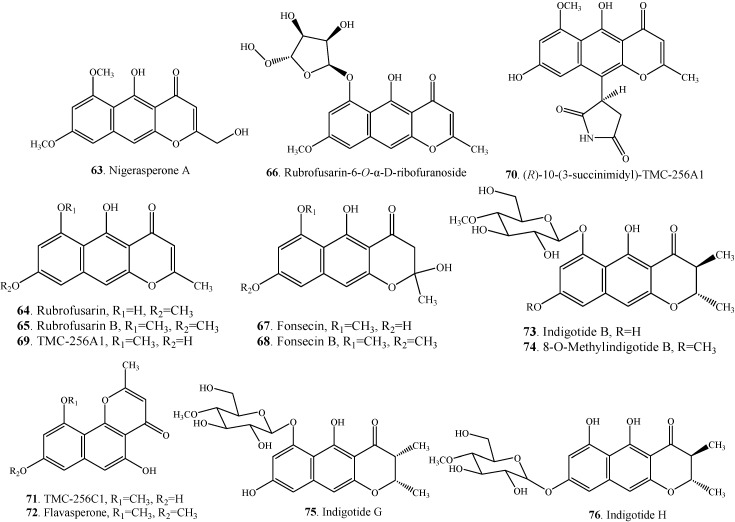
Structures of some monomeric naphtho-γ-pyrones **63**–**76** from fungi.

Chaetochromin A (**1**) was the first bis-naphtho-γ-pyrone whose biosynthesis was studied by employing the fungus *Chaetomium gracile*. Both acetate and malonate were confirmed as the precursors in the biosynthesis of chaetochromin A (**1**) by employing *Chaetomium gracile* and addition of the carbon-13 labelled precussors [51]. The proposed biosynthetic pathway (Scheme 1) of asperpyrone-type bis-naphtho-γ-pyrones in the fungus *Aspergillus niger* was outlined by Chiang *et al.* [33]. The aromatic structure of naphtho-γ-pyrones suggests that a non-reducing polyketide synthase (NR-PKS) gene with the following domain organization is likely responsible for their biosynthesis: starter unit ACP transacylase (SAT), β-ketoacyl synthase (KS), acyl transferase (AT), product template (PT), acyl carrier protein (ACP), and thiolesterase/Claisen-cyclase (TE/CLC) [33]. Linear naphtho-γ-pyrone YWA1 (**61**) was known in equilibrium with the side-chain open form (**60**). After recyclization, angular naphtho-γ-pyrone **62** could be formed in the presence of C-14 phenol group (Scheme 1). The irreversible dehydration of hemiketal from aurasperone B (**34**) produced stable dimeric naphtho-γ-pyrones fonsecinone B (**43**) and aurasperone A (**32**). Table 4 shows some monomeric naphtho-γ-pyrones such as rubrofusarin B (**65**), fonsecin (**67**), fonsecin B (**68**) and flavasperone (**72**) which were considered as the intermediates in the biosynthesis of bis-naphtho-γ-pyrones in fungi [38]. Accordingly, the structures of these proposed intermediates are shown in Figure 4.

**Table 4:** Some monomeric naphtho-γ-pyrones **63**–**76** from fungi.

	Monomeric Naphtho-γ-pyrone	Fungal Species	Reference
	Nigerasperone A (**63**)	*Aspergillus niger* EN-13	[45]
	Rubrofusarin (**64**)	*Aspergillus niger*	[38]
		Endophytic fungus *Aspergillus tubingensis* GX1-5E	[46]
	Rubrofusarin B = Heminigerone (**65**)	*Alternaria alternata*	[27]
		Endophytic fungus *Aspergillus* sp.	[34]
		Endophytic fungus *Aspergillus niger* IFB-E003	[31]
		Marine-derived fungus *Aspergillus carbonarius*	[7]
		*Aspergillus niger* var. *niger* TC 1629	[52]
		Endophytic fungus *Aspergillus tubingensis* GX1-5E	[46]
		Endophytic fungus *Aspergillus tubingensis* NRRC 4700	[36]
		Endophytic fungus *Cladosporium herbarum* IFB-E002	[44]
	Rubrofusarin-6-*O*-α-D-ribofuranoside (**66**)	Endophytic fungus *Aspergillus niger*	[8]
	Fonsecin (**67**)	*Alternaria alternata*	[27]
		Marine-derived fungus *Aspergillus carbonarius*	[7]
		*Aspergillus niger*	[38]
		*Aspergillus niger* C-433	[40]
		*Aspergillus niger* var.*niger* TC 1629	[52]
		Endophytic fungus *Aspergillus tubingensis* GX1-5E	[47]
		Endophytic fungus *Aspergillus tubingensis* NRRC 4700	[36]
	Fonsecin B = Fonsecin monomethyl ether (**68**)	*Alternaria alternata*	[27]
		*Aspergillus niger*	[38]
		*Aspergillus niger* var. *niger* TC 1629	[52]
		Endophytic fungus *Aspergillus tubingensis* NRRC 4700	[36]
	TMC-256A1 (**69**)	Marine-derived fungus *Aspergillus carbonarius*	[7]
		*Aspergillus niger* var. *niger* TC 1629	[52]
		Endophytic fungus *Aspergillus tubingensis* GX1-5E	[46]
		Endophytic fungus *Aspergillus tubingensis* NRRC 4700	[36]
	(*R*)-10-(3-succinimidyl)-TMC-256A1 (**70**)	Endophytic fungus *Aspergillus niger*	[8]
	TMC-256C1 (**71**)	*Aspergillus niger* var. *niger* TC 1629	[52]
	Flavasperone = Asperxanthon = TMC-256C2 (**72**)	Endophytic fungus *Aspergillus* sp. DCS31	[35]
		*Aspergillus* sp. FKI-3451	[4]
		Marine-derived fungus *Aspergillus carbonarius*	[7]
		*Aspergillus niger*	[38]
		*Aspergillus niger* var. *niger* TC 1629	[52]
		Endophytic fungus *Aspergillus tubingensis*	[47]
	Indigotide B (**73**)	Entomopathogenic fungus *Cordyceps indigotica*	[53]
		Sponge-derived fungus *Metarhizium anisopliae* mxh-99	[16]
	8-*O*-Methylindigotide B (**74**)	Entomopathogenic fungus *Cordyceps indigotica*	[53]
	Indigotide G (**75**)	Sponge-derived fungus *Metarhizium anisopliae* mxh-99	[16]
	Indigotide H (**76**)	Sponge-derived fungus *Metarhizium anisopliae* mxh-99	[16]

## 4. Biological Activities

Bis-naphtho-γ-pyrones have a broad-range of biological activities such as cytotoxic, antitumor and antimicrobial properties, which are outlined in Table 5.

### 4.1. Cytotoxic and Antitumor Activity

Chaetochromins A (**1**) and D (**9**) from *Chaetomium* sp. showed strong cytotoxicity with IC_50_ values ranging from 0.13 to 0.24 μg/mL in cell cultures of mouse embryo limb bud (LB) and midbrain (MB). Ustilaginoidin A (**15**) from *Ustilaginoidea virens* showed relatively weak cytotoxic activity [54]. Chaetochromins A (**1**), B (**4**), C (**8**) and D (**9**) exhibited strong cytotoxicity on KB cells by inhibiting deoxyribonucleic acid, ribonucleic acid and protein biosynthesis [2]. Further mechanism of action investigations for chaetochromin A (**1**) revealed that the ATP synthesis in mitochondria was inhibited by uncoupling oxidative phosphorylation and depressing state-3 respiration of mitochondria, which may explain their cytotoxicity and *in vivo* toxicity to animals [55].

Both ustilaginoidins D (**20**) and E (**21**) exhibited strong cytotoxicity on KB cells by inhibiting biosynthesis of nucleic acid and protein [2]. Ustilaginoidin A (**15**) also inhibited ATP synthesis in mitochondria by uncoupling oxidative phosphorylation and depressing state-3 respiration of mitochondria [55].

Cephalochromin (**11**) exhibited growth-inhibitory and apoptotic activity against human lung cancer A549 cells in a dose-dependent manner with an IC_50_ value of 2.8 μM at 48 h. Cephalochromin induced cell cycle arrest at the G0/G1 phase through down-regulation of cyclin D1, cyclin E, Cdk 2, and Cdk 4 expressions. It markedly increased the hypodiploid sub-G1 phase (apoptosis) of the cell cycle at 48 h as measured by flow cytometric analysis. Reactive oxygen species generation and loss of the mitochondrial membrane potential (MMP) were also markedly induced by cephalochromin [18]. Cephalochromin (**11**) also inhibited ATP synthesis in mitochondria by uncoupling oxidative phosphorylation and depressing state-3 respiration of mitochondria [55].

### 4.2. Antimicrobial Activity

Isochaetochromin B_2_ (**6**) and ustilaginoidin D (**20**) isolated from the sponge-derived fungus *Metarhizium anisopliae* mxh-99 exhibited anti-tubercular activity with MIC values of 50.0 μg/mL [16]. 8'-*O*-Demethylnigerone (**57**) and 8'-*O*-demethylisonigerone (**58**) from the marine-derived *Aspergillus carbonarius* also showed weak anti-tubercular activity against *Mycobacterium tuberculosis* H37Rv with MIC values of 43.0 and 21.5 μM, respectively [7].

Cephalochromin (**11**), isoustilaginoidin A (**16**), and dihydroisoustilaginoidin A (**17**) isolated from *Verticillium* sp. K-113 were active against Gram-positive bacteria (*Bacillus subtilis*, *Staphylococcus aureus*, and *Streptococcus pyogenes*) with MIC values ranging from 3 to 10 μg/mL, but not against Gram-negative bacteria and fungi [21]. Chaetochromin A (**1**), isochaetochromin A_2_ (**3**), and chaetochromin B (**4**) possessed significant antibacterial activity against *Escherichia coli*, *Staphylococcus aureus* and *Bacillus subtilis* [12]. Asperpyrone B (**28**), aurasperone A (**32**) and fonscinone A (**42**) isolated from the endophytic fungus *Aspergillus niger* IFB-E003 exhibited growth inhibition against bacteria (*Bacilllus subtilis*, *Escherichia coli* and *Pseudomonas fluorescence*) and fungi (*Trichophyton rubrum* and *Candida albicans*) with MIC values ranging from 1.9 to 31.2 μg/mL [31].

Asperpyrone A (**27**), isoaurasperone A (**33**), dianhydroaurasperone C (**36**), and fonsecinone A (**42**) from an endophytic *Aspergillus* species showed antimicrobial activities. Among them, fonsecinone A (**42**) exhibited the strongest antifungal and antibacterial activity, with MIC values of 12.5 and 25 μM, respectively [34].

Bacterial enoyl-acyl carrier protein reductase (FabI) in bacterial fatty acid synthesis has been demonstrated to be an antibacterial target [56]. Cephalochromin (**11**) inhibited FabI of *Staphylococcus aureus* and *Escherichia coli* with IC_50_ values of 1.9 and 1.8 μM, respectively [57].

### 4.3. Other Biological Activities

In addition to the cytotoxic, antitumor and antimicrobial activities of bis-nathphtho-γ-pyrones mentioned above, other biological activities include tyrosine kinase inhibition [23], HIV-1 integrase inhibition [3], calmodulin-sensitive cycle nucleotide phosphodiestease inhibition [22], triacylglycerol synthesis inhibition [14], xanthine oxidase inhibition [31], acyl-CoA:cholesterol acyltransferase inhibition [4], central nerveous system depressant effects [42], immunological activity [12], botulinum neutotoxin serotype A inhibition [57], drug resistance-reversing activity [58], and *Taq* DNA polymerase inhibition [30], which are outlined in Table 5.

**Table 5:** Biological activities of bis-naphtho-γ-pyrones from fungi.

Bis-naphtho-γ-pyrone	Biological Activity	Reference
Chaetochromin A (**1**)	Cytotoxic and antitumor activity	[2,54,55]
	Teratogenicity to mice embryo	[59]
	Inhibitory effects on nitric oxide (NO) production by activated macrophages	[60]
	Antibacterial activity	[12]
	Immunological activity	[12]
	Inhibitory activity on botulinum neurotoxin serotype A	[58]
Isochaetochromin A_1_ (**2**)	Inhibitory activity on triacylglycerol synthesis	[14]
Isochaetochromin A_2_ (**3**)	Antibacterial activity	[12]
	Immunological activity	[12]
Chaetochromin B (**4**)	Cytotoxic and antitumor activity	[2,54]
	Antibacterial activity	[12]
	Immunological activity	[12]
Isochaetochromin B_1_ (**5**)	HIV-1 integrase inhibitory activity	[3]
	Inhibitory activity on triacylglycerol synthesis	[14]
Isochaetochromin B_2_ (**6**)	Anti-tubercular activity	[16]
	HIV-1 integrase inhibitory activity	[3]
	Inhibitory activity on triacylglycerol synthesis	[14]
Oxychaetochromin B (**7**)	HIV-1 integrase inhibitory activity	[3]
Chaetochromin C (**8**)	Cytotoxic and antitumor activity	[2,54]
Chaetochromin D (**9**)	Cytotoxic and antitumor activity	[2,54]
	Impairing effects on mitochondrial respiration and structure	[61]
Chaetochromin D_1_ (**10**)	HIV-1 integrase inhibitory activity	[3]
Cephalochromin (**11**)	Cytotoxic and antitumor activity	[18,55]
	Inhibitory effects on nitric oxide (NO) production by activated macrophages	[57]
	Antimicrobial activity	[21,57]
	Inhibitory activity on calmodulin-sensitive cyclic nucleotide phosphodiestease	[22]
	Inhibitory activity on botulinum neurotoxin serotype A	[58]
Hypochromin A (**12**)	Tyrosine kinase inhibitory activity	[23]
Hypochromin B (**13**)	Tyrosine kinase inhibitory activity	[23]
SC2051 (**14**)	Tyrosine kinase inhibitory activity	[23]
Ustilaginoidin A (**15**)	Cytotoxic and antitumor activity	[54,55]
Isoustilaginoidin A (**16**)	Antimicrobial activity	[21]
Dihydroisoustilaginoidin A (**17**)	Antimicrobial activity	[21]
	Inhibitory effects on nitric oxide (NO) production by activated macrophages	[62]
Ustilaginoidin D (**20**)	Cytotoxic and antitumor activity	[2]
	Anti-tubercular activity	[16]
Ustilaginoidin E (**21**)	Cytotoxic and antitumor activity	[2]
Asperpyrone A (**27**)	Antimicrobial activity	[34]
	Inhibitory activity on *Taq* DNA polymerase	[30]
Asperpyrone B (**28**)	Antimicrobial activity	[31]
Aurasperone A (**32**)	Antimicrobial activity	[31]
	Inhibitory activity on xanthine oxidase	[31]
	Inhibitory activity on acyl-CoA:cholesterol acyltransferase	[4]
	Inhibitory activity on *Taq* DNA polymerase	[30]
Isoaurasperone A (**33**)	Antimicrobial activity	[34]
Dianhydro-aurasperone C (**36**)	Antibacterial activity	[34]
	Drug resistance-reversing activity	[59]
Aurasperone D (**37**)	Central nervous system depressant effects	[42]
	Inhibitory activity on acyl-CoA:cholesterol acyltransferase	[4]
Fonscinone A (**42**)	Antimicrobial activity	[31,34]
	Inhibitory activity on *Taq* DNA polymerase	[30]
8'-*O*-Demethylnigerone (**57**)	Anti-tubercular activity	[7]
8'-*O*-Demethylisonigerone (**58**)	Anti-tubercular activity	[7]

## 5. Conclusions and Future Perspectives

About 59 fungal bis-naphtho-γ-pyrones have been investigated in the past few decades. Some of them display diverse bioactivities, especially cytotoxic, antitumor and antimicrobial activities. The remaining bis-naphtho-γ-pyrones produced by fungi and their bioactivities need to be further studied. In recent years, an increasing number of bis-naphtho-γ-pyrones have been isolated from endophytic fungi [3,10,20] and marine-derived fungi [7,16,23]. These fungi could be the rich sources of biologically active metabolites that are indispensable for medicinal and agricultural applications [1,63,64,65,66]. In most cases, biological activities, structure-activity relationships, and mode of action of bis-naphtho-γ-pyrones have been only primarily investigated and need to be studied in detail. The potential applications of bis-naphtho-γ-pyrones as antitumor agents, antimicrobials, and antivirus agents as well as their promising bioactivities have led to considerable interest within the pharmaceutical community. Chemical syntheses have been achieved for a few bioactive bis-naphtho-γ-pyrones such as ustilaginoidin A (**15**) [67] and nigerone (**55**) [68,69]. After comprehensive understanding of biosynthetic pathways of some bis-naphtho-γ-pyrones in the next few years, we can effectively not only increase yields of the bioactive bis-naphtho-γ-pyrones (*i.e.*, cephalochromin, isochaetochromins B_1_ and B_2_), but also prevent biosynthesis of some toxic bis-naphtho-γ-pyrones (*i.e*., ustilaginoidins A–J) [70]. In addition, the physiological and ecological roles of the bis-naphtho-γ-pyrones in fungi need to be clarified in detail [71,72].
